# Effects of Fractionation and Combinatorial Evaluation of *Tamarindus indica* Fractions for Antibacterial Activity

**DOI:** 10.3390/molecules16064818

**Published:** 2011-06-09

**Authors:** Uchechukwu U. Nwodo, Christian U. Iroegbu, Augustine A. Ngene, Vincent N. Chigor, Anthony I. Okoh

**Affiliations:** 1 Applied and Environmental Microbiology Research Group (AEMREG), Department of Biochemistry and Microbiology, University of Fort Hare, Private Bag X1314, Alice 5700, South Africa; Email: vchigor@ufh.ac.za (V.N.C.); aokoh@ufh.ac.za (A.I.O.); 2 Department of Microbiology, University of Nigeria, Nsukka, Nigeria; Email: chrisuiroegbu@yahoo.com; 3 Department of Veterinary Medicine, University of Nigeria, Nsukka, Nigeria; Email: austinearinze@yahoo.com

**Keywords:** combinatorial assay, thin layer chromatography, phytochemistry, subfraction, antibacterial activity, solvent system

## Abstract

Six fractions, named TiA – TiF, were obtained by fractionating the crude ethanol extract of the stem bark of *Tamarindus indica* using column chromatographic techniques. On TLC, fraction TiB showed five bands, TiC three bands, while TiD and TiE showed two bands each. TiC, TiD and TiE were re-eluted with different solvent systems to yield two fractions each, while TiB yielded four. These subfractions were designated B1-B4; C1-C2; D1-D2 and E1-E2, respectively. Tannins, flavonoids and alkaloids, among other components, were detected, albeit in different proportions with respect to fractions and subfractions and were compartmentalized with respect to the solvent systems used. The *in vitro* antibacterial activity of fractions and subfractions was tested separately and in combinations using the agar well diffusion technique. The susceptibly of test strains (expressed as %) were: 83.3% (TiA and TiB), 75.0% (crude extract and TiC), 66.7% (TiD), 50.0% (TiE) and 16.7% (TiF) when used singly, whereas in combination, the corresponding susceptibilities were 100% (CE), 83.3% (DE), 66.7% (AB, AF, BC, BD, DE and EF), 50% (AC and CD), 33.3% (BE and BF) and 16.7% (AD) against Gram negative bacteria strains and 100% (EF), 80% (DE), 60% (AB, BC and CE), 40% (AC, BD, BF, CF and DF) and 20% (AE, AF, BE and CD) against Gram positive strains. Percentage susceptibility with combinatorial use of re-fractions ranged from 85.7–57.1% and 60–40% against Gram negative and positive strains (TiB subfractions), respectively, 100–85.7% and 40–0% against Gram negative and positive strains (TiC, TiD and TiE sub-fractions).

## 1. Introduction

Multiple targets, minor side-effects, low resistance due to selective pressure of infective agents and low cost are some of the important properties of medicinal plants [1,2,3,4] that have led to the investigation of their antibacterial activity [5,6,7,8,9,10]. In the Western culture, where herbal medicine seems to have been relegated by the preference for synthetic drugs, there is currently a rethinking and resurgence of herbal remedies. The new direction has been necessitated by the high rates of resistance to antimicrobial drugs and the apparent residual toxicity of many synthetic drugs. Medicinal plant materials are generally considered safe and understandably so as most of them are easily metabolized in humans and animals. With advances in chemistry and particularly, extraction methods, active principles of such medicinal plants have been isolated and studied. These became templates for the synthesis of analogue drugs and thus heralded the advent of synthetic drugs [11]. 

Most of the active principles isolated turned out to be secondary metabolites; *i.e.*, products not necessarily required by plants but produced, excreted and stored in such locations as the stem bark or root as waste or as protective deposits [12]. Indeed, several functions have been ascribed to secondary metabolites *in situ*. For example, some secondary metabolites are toxins used to deter predation, while others may be pheromones used to attract insects for pollination. Phytoalexins protect against bacterial and fungal attacks while allelochemicals inhibit rival plants that are competing for soil and light. Plants up-regulate and down-regulate their biochemical paths in response to the local mix of herbivores, pollinators and microorganisms [12]. Also, it is generally believed that fractionation of plant extracts and purification of the active principles would optimize their potencies. However, in some cases fractionation has been found to extend the spectrum of activity of plant extracts [10,13], while in others it was found to reduce the spectrum of activity [10], depending on whether certain constituents of the crude extract interact antagonistically, synergistically or additively when used in combination. *Tamarindus indica*, the subject of this report, is reputed as a folklore herbal remedy for several ailments as well as source of food and beverage for man and animals in various parts of the World. In this study, the column chromatographic fractions of the ethanol extracts were evaluated in combination antimicrobial studies.

## 2. Results and Discussion

### 2.1. Results

**Figure 1:**
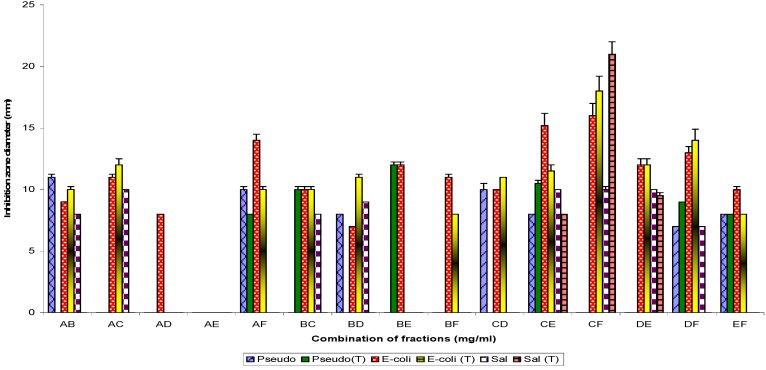
Antibacterial susceptibility pattern of the Gram negative bacterial test strains to combination of fractions Ti (A-F).

Column chromatographic fractionation of the ethanol extract of *T. indica* stem bark yielded (in order of elution) the fractions designated as TiA, TiB, TiC, TiD, TiE and TiF. TiA and TiF each showed a single band by TLC, whereas TiB, TiC, TiD and TiE showed five, three, two and two bands, respectively. On refractionation of fractions with multiple bands TiB yielded four distinct fractions and each gave a single band on TLC. These were designated B1, B2, B3 and B4, while TiC, TiD and TiE yielded two fractions each that were similarly designated C1 and C2, D1and D2, E1 and E2 respectively [10]. The phytoconstituents of all the fractions and subfractions (TiA, TiB, TiC, TiD, TiE, TiF, B1, B2, B3, B4, C1, C2, D1, D2, E1 and E2) and their individual antibacterial activity has been reported in [10]. Gram negative bacteria test strains showed susceptibility to the pairwise combinatorial use of the fractions and to each combination as follows: 100% (CE), 83.0% (DE), 66.7% (AB, AF, BC, BD, CF, DF and EF) 50% (AC and CD), 33.3% (BE and BF) and 16.7% (AD), respectively (Figure 1).

**Figure 2:**
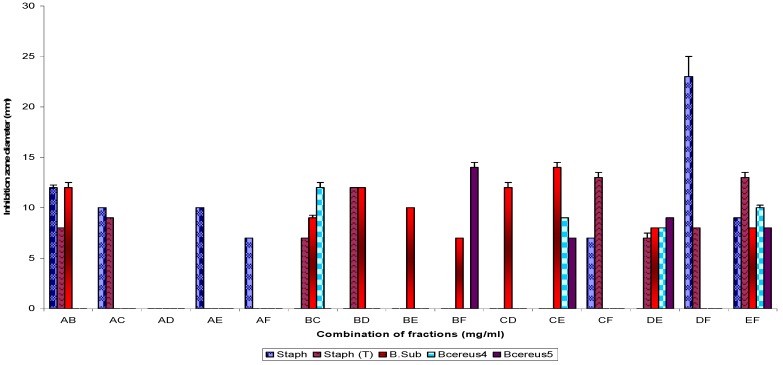
Antibacterial susceptibility pattern of the Gram positive bacterial test strains to combination of fractions Ti (A-F).

**Figure 3:**
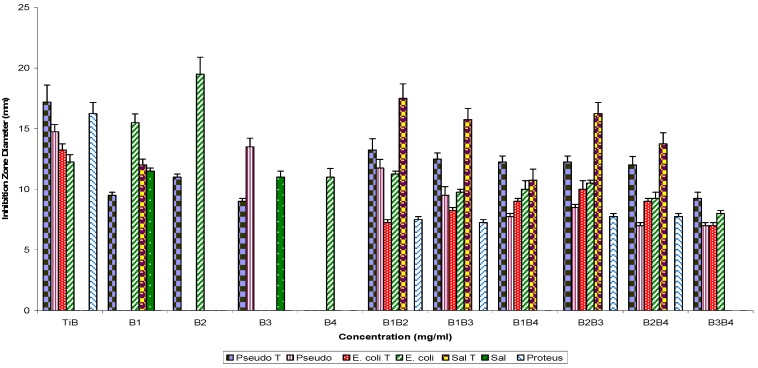
Antibacterial susceptibility pattern of Gram negative bacterial test strains to fraction TiB, its refraction products singly and in combination.

The Gram positive bacterial strain (Figure 2) test percentage susceptibilities were: 100% (EF), 80% (DE), 60% (AB, BC and CE), 40% (AC, BD, BF, CF and DF) and 20% (AE, AF, BE and CD). Similarly, use of TiB subfractions alone showed Gram negative test susceptibility as follows: 57% (B1), 42.9% (B3), 28.6% (B2), and 14.2% (B4). When used in combination the following percentage activities were recorded: 85.7% (B1B2, B1B3, B1B3 and B2B4), 71.4% (B1B4) and 57.1% (B3B4) respectively; the control (TiB) showed 71.4% activity (Figure 3).

**Figure 4:**
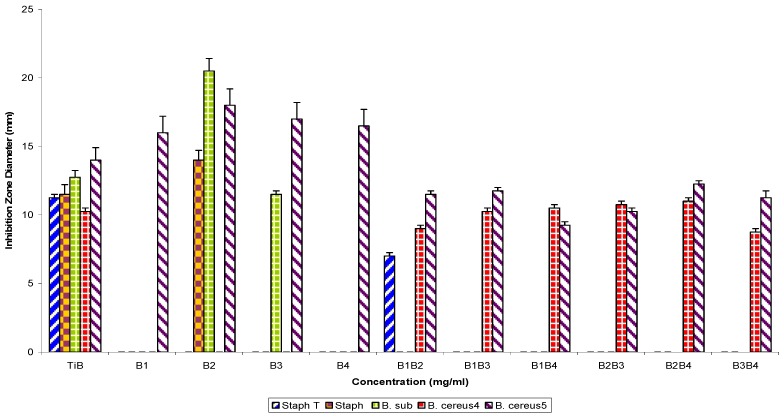
Antibacterial susceptibility pattern of Gram positive bacterial test strains to fraction TiB, its refraction products singly and in combination.

Furthermore, assaying the same fractions singly against Gram positive bacterial test strains showed percentage susceptibilities of 60% (B2), 40% (B3), and 20% (B1 and B4), respectively, and in combinatorial use, susceptibilities were 60% (B1B2) and 40% (B1B3, B1B4, B2B3, B2B4 and B3B4) each, and 100% susceptibility to the control (Figure 4).

**Figure 5:**
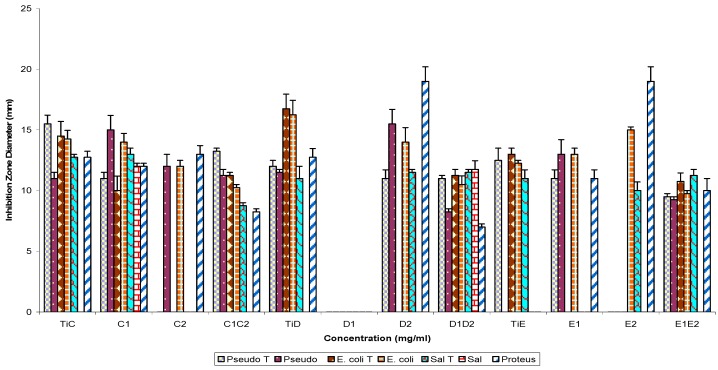
Antibacterial susceptibility pattern of Gram negative bacterial test strains to fraction TiC, TiD and TiE, refraction products singly and in combination.

**Figure 6:**
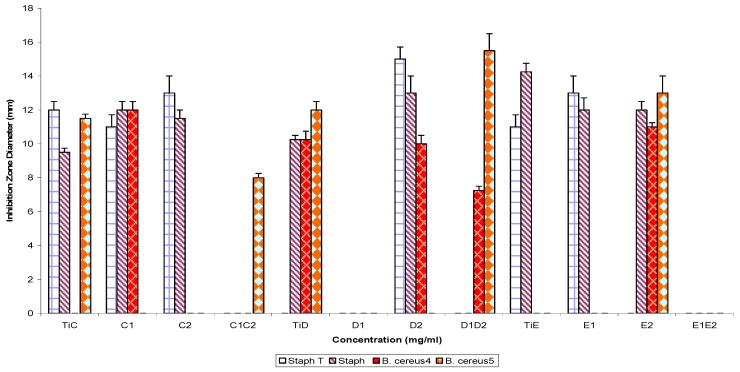
Antibacterial susceptibility pattern of Gram positive bacterial test strains to fraction TiC, TiD and TiE, refraction products singly and in combination.

The spectra of activity of subfractions C1, C2, D1, D2, E1, E2 and their parent fractions (controls) tested singly and in combination against the Gram negative and Gram positive test strains are shown in Figure 5 and Figure 6. The assay of sub-fractions singly and in combination against test Gram negative bacteria strains showed the following percentage susceptibilities: 100% (C1), 42.9% (C2), 85.7% (C1C2), 85.7% (TiC-control); 0% (D1), 71.4% (D2), 100% (D1D2), 85.7% (TiD-control); 57.1% (E1), 41.9% (E2), 85.7% (E1E2) and 57.1% (TiE-control), respectively. On the other hand, the Gram positive bacterial strains showed percentage susceptibilities of 60% (C1), 40% (C2), 20% (C1C2), 60% (TiC-control); 0% (D1), 60% (D2), 40% (D1D2), 60% (TiD-control); 40% (E1), 60% (E2) 85.7% (E1E2) and 40% (TiE-control) respectively. 

Pairwise use of parent fractions (Figure 1 and Figure 2) showed inhibition zone diameter ranges of 21.0 ± 1.2 to 0.0 ± 0.0 (p = 0.05) against the Gram negative bacterial strains and 23.2 ± 2.1 to 0.0 ± 0.0 against the Gram positive bacterial test strains. Subfractions, similarly, showed inhibition zone diameter ranges of 19.50 ± 1.4 to 7.0 ± 0.25 mm (TiB and subfraction), 15.0 ± 1.2 to 8.25 ± 0.25 mm (TiC and subfraction), 19.0 ± 1.2 to 7.0 ± 0.25 mm (TiD and subfraction) and 15.0 ± 0.25 to 9.25 ± 0.25 mm (TiE and subfractions) against the Gram negative bacterial test strains, while for the Gram positive bacteria test strains, inhibition zone diameter ranged as follows: 20.5 ± 0.91 to 7.0 ± 0.25 mm (TiB and subfractions), 12.0 ± 0.5 to 8.0 ± 0.25 mm (TiC and subfractions), 15.5 ± 1.0 to 7.25 ± 0.25 mm (TiD and subfractions) and 14.25 ± 0.5 to 11.0 ± 0.25 mm (TiE and subfractions).

### 2.2. Discussion

Fractionation/purification of plant extracts may result in improved activity or loss of activity [10]. The range and type of susceptible organism varied with fraction, which is expected as active compounds move into different chromatographic fractions. To buttress this concept, assaying parent fractions (TiA-TiF) in pairwise combinations against both Gram negative and positive strains showed uneven antibacterial activities of the combinations and varied susceptibilities of the test strains. With reference to spectrum of activity, CE combination ranked highest against the Gram negative test strains and DE against Gram positives. However, with reference to susceptibilities of test strains, DF showed higher activity with *Salmonella kintambo* SSRL 113, *E. coli* ATCC11775 and *E. coli* (clinical strain) showing inhibition zone diameters of 21.2 ± 0.9, 18.3 ± 0.71 and 15.6 ± 0.5 (mm), respectively. DF, likewise, showed the highest inhibition zone diameter of 24.0 ± 1.0 mm against *Staphylococcus aureus* (clinical strain). Combinatorial use of fractions and subfractions resulted severally in enhanced, reduced or loss of activity. For example, when TiB was re-fractionated, one of its subfractions (B4) showed no activity against *Pseudomonas aeruginosa* ATCC 10145 and B3 showed no activity against clinical strain of *Ps. aeruginosa* compared to parent fraction TiB, which should be expected, as components are separated from one another suggesting that components were composed of different constituents. This is in line with the views of Marjorie [12] who observed that combination of secondary metabolites enhanced activity of the combined agents and spectrum of susceptible organisms. With reference to *E. coli* susceptibility, subfractions of TiB - B1, B2, B3, and B4 - lost activities against the typed strain, while only B3 had activity against the clinical strain. This is a clear example of the situation were purification has led to loss of activity, which suggests that components of the sub-fraction acted either in synergy or additively to produce the activity shown by TiB. The exception is B3, which showed activity singly against clinical *E. coli* isolate indicating that it has a singly active constituent. Situations where purification has led to loss of activity have been reported [14]. Other variations in activity of fractions and subfractions in comparison with the parent may simply represent variations in strain susceptibility.

A converse situation was observed with subfractions of TiC, C1 and C2, whereby C1 had activity against *Salmonella sp*. but TiC, the parent fraction, and subfraction C2 had none, indicating that fractionation restored activity that was lacking in TiC. This suggests that there was a component in TiC contained in C2, which antagonized the active principles that now separated into Cl. This was confirmed when C1 and C2 where combined and activity against *Salmonella sp.* was lost again. A curious situation arose when TiD which did not have activity against *Salmonella sp.* and when fractioned into D1 and D2 the latter two fractions still did not show activity against *Salmonella sp.*; but when D1 and D2 were combined they showed activity against *Salmonella sp.* It was inferred that some other principles in TiD antagonized the D1D2 combination *in situ* in the TiD fraction; otherwise, components of D1 and D2 may have acted synergistically to produce activity. The inferred antagonistic principle(s) in TiD, could not be accounted for since it could have been lost while eluting independent of D1 and D2. This shows that the interactions amongst constituents of plant materials may be more complex than otherwise assumed. Sub-fractions of TiE showed interactions similar to TiC where TiE was not active against test Salmonella strain but when fractionated into E1 and E2, all two sub-fractions showed activity against *Salmonella sp.* Thus, E1 and E2 may be thought to antagonize one another in the TiE fraction. However, when these two fractions were combined, they showed activity unlike the parent TiE. Again, it may be assumed that the antagonistic principle in TiE was independent of E1 and E2 and was lost in the process of fractionation.

## 3. Experimental

### 3.1. Plant Materials

The stem bark of the plant evaluated was obtained from the woods of More in Sokoto South Local Government Area, Sokoto State of Nigeria. The plant species was identified taxonomically at the herbarium unit of the Department of Botany, University of Nigeria, Nsukka, where a voucher specimen was deposited.

### 3.2. Extractions of the Plant Materials

Fresh stem bark of *Tamarindus indica* were rinsed thoroughly in running tap water, chopped to tiny pieces and air dried at room temperature (~28 °C). The dried stem bark was pulverised into powder. 50.0 g of the milled material was macerated into 200 mL of absolute ethanol (Sigma-Aldrich) and left to stand for about four hours. The preparation was filtered through Whatman No. 1 filter paper and filtrate concentrated to dryness in a steady air current. The extract was stored in sterile containers at a temperature of 4 °C until further use.

### 3.3. Fractionation of Extract

The ethanol extract of the stem bark of *Tamarindus indica* were re-constituted in absolute ethanol and spotted on analytical TLC (silica gel G_600_ 0.25 mm thickness) and the solvent systems developed. Fractionation and re-fractionations of the extracts were carried out using standard methods [10].

### 3.4. Phytochemical Screening

The crude ethanolic extract of *T. indica* stem bark, initial fractions (TiA - TiF) and products of re-fractions were screened for the presence of alkaloids, saponins, tannins, anthraquinones, glycosides, flavonoids, reducing sugar, carbohydrates and sterol in accordance to standard phytochemical methods [15,16].

### 3.5. Test Bacterial Strains

Clinical isolates of *Staphylococcus aureus* from a case of sexually transmitted infection (STI) and *Escherichia coli* from a case of gastroenteritis were collected from the Clinical Diagnostic Laboratory, Department of Microbiology, University of Nigeria, Nsukka. *Bacillus cereus* (NRRL 14724 and NRRL 14725) were obtained from the Bacteriology Unit of the same department. Type cultures of *Pseudomonas aeruginosa* (ATCC 10145), *Escherichia coli* (ATCC 11775), *Bacillus subtilis* (ATCC 6051), and *Staphylococcus aureus* (ATCC 12600) were obtained from the Bioresources Development and Conservation Project (BDCP), Nsukka. *Salmonella kintambo* (SSRL 113) was supplied by the Veterinary Microbiology and Pathology Laboratory of the University of Nigeria, Nsukka. Each test bacterial strain was purified by re-isolating severally on Mueller Hinton agar (Oxoid) and emergent discrete colonies picked and identify reaffirmed after characterization by standard bacteriological methods [17]. Stock cultures were maintained in nutrient agar slants at 4 °C.

### 3.6. Evaluation of Fractions and Re-Fractions for Antibacterial Activity

Fractions and re-fractions were assayed for antibacterial activity singly and in combination using the agar well diffusion technique. Standardized inoculum was adjusted to 1.0 × 10^6^ CFU/mL for Gram-positive bacteria and 5 × 10^5^ CFU/mL for Gram-negative bacteria [18]. One hundred microliter of the test bacterial strain suspension was seeded evenly on to sterile Muller Hinton agar plates so as to achieve a confluent growth. The plates were allowed to dry and a sterile cork borer of diameter 6.0 mm was used to bore wells in the agar plates. The extracts were reconstituted with sterile distilled water to a concentration of 62.5 mg/mL. Subsequently, a 100 µL volume of the extracts were introduced in triplicate wells into the MHA cultures. The plates were allowed to stand for about 2 hours for diffusion to take place and then incubated at 37 °C for 24 h. The inhibition zone diameter was recorded to the nearest mm.

## 4. Conclusions

This study highlights some of the implications of purification of plant fractions which includes varied activity of the resultant fractions to otherwise susceptible test bacteria. This observation was quite evident as some bacterial strains lost susceptibility and others showed improved susceptibility to the purification products. Hence, purification has been shown to be an important tool in the screening of novel antibacterial agents. However, a well guided approach towards selectively isolating plant active compounds which are specific in activity against test strains is imperative; as a result quality control is necessary in this kind of process. The intercombination of the products of subfractions from different generic groups of the same parent fraction and their subsequent loss in activity may however, require further enquiry into what precisely happened. Regardless of the mechanism(s) in play, this study showed that fractionation led to loss of activity but not in all cases. In some instances, activity was restored when the fractions where recombined while in other cases previously non susceptible test strains became susceptible. Hence, bioassay guided plant extract purification is imperative in the search for novel antimicrobials. *Tamarindus indica* has shown good potential as source of novel antimicrobial agent as vast array of its fractions were potent against test bacteria strains.
